# ECLAPTE: *Effective Closure of LAParoTomy in Emergency*—2023 World Society of Emergency Surgery guidelines for the closure of laparotomy in emergency settings

**DOI:** 10.1186/s13017-023-00511-w

**Published:** 2023-07-26

**Authors:** Simone Frassini, Lorenzo Cobianchi, Paola Fugazzola, Walter L. Biffl, Federico Coccolini, Dimitrios Damaskos, Ernest E. Moore, Yoram Kluger, Marco Ceresoli, Raul Coimbra, Justin Davies, Andrew Kirkpatrick, Isidoro Di Carlo, Timothy C. Hardcastle, Arda Isik, Massimo Chiarugi, Kurinchi Gurusamy, Ronald V. Maier, Helmut A. Segovia Lohse, Hans Jeekel, Marja A. Boermeester, Fikri Abu-Zidan, Kenji Inaba, Dieter G. Weber, Goran Augustin, Luigi Bonavina, George Velmahos, Massimo Sartelli, Salomone Di Saverio, Richard P. G. Ten Broek, Stefano Granieri, Francesca Dal Mas, Camilla Nikita Farè, Jacopo Peverada, Simone Zanghì, Jacopo Viganò, Matteo Tomasoni, Tommaso Dominioni, Enrico Cicuttin, Andreas Hecker, Giovanni D. Tebala, Joseph M. Galante, Imtiaz Wani, Vladimir Khokha, Michael Sugrue, Thomas M. Scalea, Edward Tan, Mark A. Malangoni, Nikolaos Pararas, Mauro Podda, Belinda De Simone, Rao Ivatury, Yunfeng Cui, Jeffry Kashuk, Andrew Peitzman, Fernando Kim, Emmanouil Pikoulis, Gabriele Sganga, Osvaldo Chiara, Michael D. Kelly, Ingo Marzi, Edoardo Picetti, Vanni Agnoletti, Nicola De’Angelis, Giampiero Campanelli, Marc de Moya, Andrey Litvin, Aleix Martínez-Pérez, Ibrahima Sall, Sandro Rizoli, Gia Tomadze, Boris Sakakushev, Philip F. Stahel, Ian Civil, Vishal Shelat, David Costa, Alain Chichom-Mefire, Rifat Latifi, Mircea Chirica, Francesco Amico, Amyn Pardhan, Vidya Seenarain, Nikitha Boyapati, Basil Hatz, Travis Ackermann, Sandun Abeyasundara, Linda Fenton, Frank Plani, Rohit Sarvepalli, Omid Rouhbakhshfar, Pamela Caleo, Victor Ho-Ching Yau, Kristenne Clement, Erasmia Christou, Ana María González Castillo, Preet K. S. Gosal, Sunder Balasubramaniam, Jeremy Hsu, Kamon Banphawatanarak, Michele Pisano, Toro Adriana, Altomare Michele, Stefano P. B. Cioffi, Andrea Spota, Fausto Catena, Luca Ansaloni

**Affiliations:** 1 University of Pavia, Corso Str. Nuova, 65, 27100 Pavia, Italy; 2 Unit of General Surgery I, Fondazione I.R.C.C.S. Policlinico San Matteo, Viale Camillo Golgi, 19, 27100 Pavia, Italy; 3 Department of Emergency and Trauma Surgery, Scripps Clinic Medical Group, La Jolla, CA USA; 4 General, Emergency and Trauma Surgery Department, Pisa University Hospital, Pisa, Italy; 5 General and Emergency Surgery, Royal Infirmary of Edinburgh, Edinburgh, UK; 6 Ernest E Moore Shock Trauma Center at Denver Health, Denver, CO USA; 7 Division of General Surgery, Rambam Health Care Campus, Haifa, Israel; 8 General Surgery, Monza University Hospital, Monza, Italy; 9 Riverside University Health System Medical Center, Comparative Effectiveness and Clinical Outcomes Research Center - CECORC, Claremont, CA USA; 10 Cambridge Colorectal Unit, Addenbrooke’s Hospital, Cambridge, UK; 11 Departments of Surgery and Critical Care Medicine, University of Calgary, Calgary, Canada; 12 Department of Surgical Sciences and Advanced Technologies, General Surgery Unit, Cannizzaro Hospital, Catania, Italy; 13 Department of Surgical Sciences, Nelson R Mandela School of Clinical Medicine, University of KwaZulu-Natal, Durban, 4001 South Africa; 14 Trauma and Burns Services, Inkosi Albert Luthuli Central Hospital, Mayville, 4058 South Africa; 15 Division of General Surgery, School of Medicine, Istanbul Medeniyet University, Istanbul, Turkey; 16 Division of Surgery and Interventional Science, Hampstead Campus, University College London, London, UK; 17 Department of Surgery, Harborview Medical Centre, University of Washington, Seattle, USA; 18 II Cátedra de Clínica Quirúrgica, Hospital de Clínicas, Universidad Nacional de Asunción, San Lorenzo, Paraguay; 19 Department of Surgery, Erasmus University Medical Centre, Rotterdam, The Netherlands; 20 Department of Surgery, Amsterdam University Medical Center, University of Amsterdam, 1105AZ Amsterdam, The Netherlands; 21 The Research Office, College of Medicine and Health Sciences, United Arab Emirates University, Al-Ain, UAE; 22 Los Angeles County + USC Medical Center, 2051 Marengo Street, Room C5L100, Los Angeles, CA 90033 USA; 23 Department of General Surgery, Royal Perth Hospital, University of Western Australia, Perth, Australia; 24 Department of Surgery, University Hospital Centre Zagreb, Zagreb, Croatia; 25 Division of General Surgery, Department of Biomedical Science for Health, I.R.C.C.S. Ospedale Galeazzi – Sant’Ambrogio, Milan, Italy; 26 Division of Trauma, Emergency Surgery, and Surgical Critical Care, Massachusetts General Hospital and Harvard Medical School, Boston, MA USA; 27 Department of Surgery, Macerata Hospital, Macerata, Italy; 28 Unit of General Surgery, San Benedetto del Tronto Hospital, av5 Asur Marche, San Benedetto del Tronto, Italy; 29 Department of Surgery, Radboud University Medical Center, Nijmegen, The Netherlands; 30 General Surgery Unit, ASST Vimercate, Via Santi Cosma E Damiano, 10, 20871 Vimercate, Italy; 31 Department of Management, Università Ca’ Foscari, Dorsoduro 3246, 30123 Venezia, Italy; 32 Department of General and Thoracic Surgery, University Hospital of Giessen, Giessen, Germany; 33 Department of Digestive and Emergency Surgery, S. Maria Hospital Trust, Terni, Italy; 34 Trauma Department, University of California, Davis, Sacramento, CA USA; 35 Government Gousia Hospital, Srinagar, India; 36 Department of Emergency Surgery, City Hospital, Mozyr, Belarus; 37 Donegal Clinical Research Academy Emergency Surgery Outcome Project, Letterkenny University Hospital, Donegal, Ireland; 38 Cowley Shock Trauma Center at the University of Maryland, Baltimore, MD USA; 39 Department of Surgery, MetroHealth Medical Center Campus, Case Western Reserve University, Cleveland, OH 44109 USA; 40 Third Department of Surgery, Attikon University Hospital, 15772 Athens, Greece; 41 Department of Surgical Science, Cagliari State University, Cagliari, Italy; 42 Department of Emergency Surgery, Centre Hospitalier Intercommunal de Villeneuve-Saint-Georges, Villeneuve-Saint-Georges, France; 43 Virginia Commonwealth University, Richmond, VA USA; 44 Department of Surgery, Tianjin Nankai Hospital, Nankai Clinical School of Medicine, Tianjin Medical University, Tianjin, China; 45 Department of Surgery, Sackler School of Medicine, Tel Aviv University, Tel Aviv, Israel; 46 Division of Trauma and General Surgery, Department of Surgery, University of Pittsburgh Medical Center, Pittsburgh, PA USA; 47 Denver Health Medical Center, University of Colorado Anschutz Medical Center, Aurora, CO USA; 48 Medical School, National and Kapodistrian University of Athens (NKUA), Athens, Greece; 49 Fondazione Policlinico Universitario A.Gemelli IRCCS, Università Cattolica, Rome, Italy; 50 Trauma Center and Emergency Surgery, ASST Grande Ospedale Metropolitano Niguarda, Milan, Italy; 51 Department of General Surgery, Albury Hospital, Albury, Australia; 52 Department of Trauma, Hand and Reconstructive Surgery, University Hospital Frankfurt, Frankfurt, Germany; 53 Department of Anesthesia and Intensive Care, Parma University Hospital, Parma, Italy; 54 Anesthesia and Intensive Care Unit, Ospedale M Bufalini, Cesena, Italy; 55 Service de Chirurgie Digestive et Hépato-Bilio-Pancréatique, Hôpital Henri Mondor, Université Paris Est, Créteil, France; 56 Division of General Surgery, I.R.C.C.S. Ospedale Galeazzi-Sant’Ambrogio, University of Insubria, Varese, Italy; 57 Medical College of Wisconsin, Milwaukee, WI USA; 58 AI Medica Hospital Center / Immanuel Kant Baltic Federal University, Kaliningrad, Russia; 59 Faculty of Health Sciences, Valencian International University (VIU), Valencia, Spain; 60 Department of General Surgery, Military Teaching Hospital, Hôpital Principal Dakar, Dakar, Senegal; 61 Hamad General Hospital, Doha, Qatar; 62 Department of Surgery, Tbilisi State Medical University, Tbilisi, Georgia; 63 RIMU/University Hospital St George, Plovdiv, Bulgaria; 64 Department of Orthopedic Surgery and Neurosurgery, Denver Health Medical Center, University of Colorado School of Medicine, Denver, CO USA; 65 Trauma Service, Auckland City Hospital, Auckland, New Zealand; 66 Tan Tock Seng Hospital, Singapore, Singapore; 67 Department of General y Digestive Surgery, “Dr. Balmis” Alicante General University Hospital, Alicante, Spain; 68 Faculty of Health Sciences, University of Buea, Buea, Cameroon; 69 College of Medicine, University of Arizona, Tucson, AZ USA; 70 Service de Chirurgie Digestive, Centre Hospitalier Universitaire Grenoble Alpes, Grenoble, France; 71 Department of Traumatology, John Hunter Hospital, University of Newcastle, Newcastle, NSW Australia; 72 Bunbury Hospital, Bunbury, WA 6230 Australia; 73 Acute Surgical Unit, Department of General Surgery, Fiona Stanley Hospital, Murdoch, WA Australia; 74 State Major Trauma Unit, Royal Perth Hospital, Wellington Street, Perth, Australia; 75 General Surgery, Monash Medical Centre, Monash Health, Melbourne, VIC Australia; 76 Department of Colorectal Surgery, Logan Hospital, Meadowbrook, QLD Australia; 77 Maitland Private Hospital, East Maitland, Newcastle, NSW Australia; 78 Chris Hani Baragwanath Hospital, Soweto, South Africa; 79 Nambour Selangor Private Hospital, Sunshine Coast University Private Hospital, Birtinya, QLD Australia; 80 Sir Charles Gairdner Hospital, Nedlands, WA Australia; 81 Department of Surgery, Nepean Hospital, Penrith, NSW 2751 Australia; 82 Departamento de Cirugía, Universidad Autónoma de Barcelona, Barcelona, Spain; 83 Department of General Surgery, Nepean Hospital, Sydney, NSW Australia; 84 Department of Trauma, Westmead Hospital, The University of Sydney, Sydney, NSW Australia; 85 Division of Trauma, Siriraj Hospital, Bangkok, Thailand; 86 General and Emergency Surgery, ASST Papa Giovanni XXIII, Bergamo, Italy; 87 General Surgery, Augusta Hospital, Augusta, Italy; 88 Acute Care Surgery Unit, Department of Surgery and Trauma, Maurizio Bufalini Hospital, Cesena, Italy

**Keywords:** Laparotomy closure, Midline incision, Emergency, Abdominal wall incision, Closure technique, Incisional hernia, Wound dehiscence, Wound complications

## Abstract

Laparotomy incisions provide easy and rapid access to the peritoneal cavity in case of emergency surgery. Incisional hernia (IH) is a late manifestation of the failure of abdominal wall closure and represents frequent complication of any abdominal incision: IHs can cause pain and discomfort to the patients but also clinical serious sequelae like bowel obstruction, incarceration, strangulation, and necessity of reoperation. Previous guidelines and indications in the literature consider elective settings and evidence about laparotomy closure in emergency settings is lacking. This paper aims to present the World Society of Emergency Surgery (WSES) project called ECLAPTE (Effective Closure of LAParoTomy in Emergency): the final manuscript includes guidelines on the closure of emergency laparotomy.

## Background

An appropriate incision is fundamental to performing any surgical procedure. The choice of incision in the case of laparotomy depends on the anatomical site of interest, the kind of setting, and the surgeon’s preference. In the case of emergency settings, laparotomy incisions allow rapid and easy access to the peritoneal cavity. However, incisional hernia (IH) represents a frequent complication of any abdominal wall incision.

IHs are defined as a late manifestation of failure of the abdominal fascia closure after surgical incisions [1]. The estimated incidence of IHs following major abdominal surgery ranges from 2 to 40% across studies considering both elective and emergency procedures [2]. Patient and wound factors contribute to the risk of developing an IH, but the setting—elective versus emergency—and the surgical technique seem to be an adjunctive factor for the development of these complications [2–4].

IHs can cause discomfort to the patients resulting in work and physical activities restriction, but their most redoubtable complications can include pain, deformity, bowel obstruction, incarceration, strangulation, and the necessity of both hospital readmission and reoperation marked with higher morbidity [5, 6]. Prevention of IHs is, therefore, crucial.

Several conditions contribute to the risk of developing an IH. Surgical aspects—such as the site of the incision, closure technique, suture material, and postoperative treatment—are well-described factors contributing to IHs occurring within the first two years after surgery [7–9]. Some other conditions not determined by surgeons and patients’ factors are relevant contributions to the risk of primary abdominal closure failure and hence incisional hernias [9, 10]. According to recent literature, a definition of high-risk patients for IH development has been described: patients with diabetes, chronic pulmonary disease, smoking, obesity, immunosuppression, surgical site infection (e.g., contaminated superficial fields), and previous abdominal surgery are at high risk of developing incisional hernias [11, 12].

In 2015, the European Hernia Society (EHS) published the first version of guideline statements with indications for the closure of abdominal wall incisions [13]. After that, systematic reviews and meta-analyses have been published trying to address these knowledge and evidence gaps [14]: an updated version of guidelines for the closure of midline incisions from EHS and American Hernia Society has been recently published. It aims to provide an up-to-date, complete point-of-view on this topic [15]. However, considering the heterogeneity of clinical scenarios in which a laparotomy could be performed, there are some concerns about these recent guidelines. The guidelines did not consider indications for laparotomy in emergency settings. Laparotomy is still particularly important in the emergency setting for trauma, intra-abdominal sepsis management, and other acute abdominal conditions. Therefore, evidence-based guidelines on emergency laparotomy were clearly necessary.

**Fig. 1:**
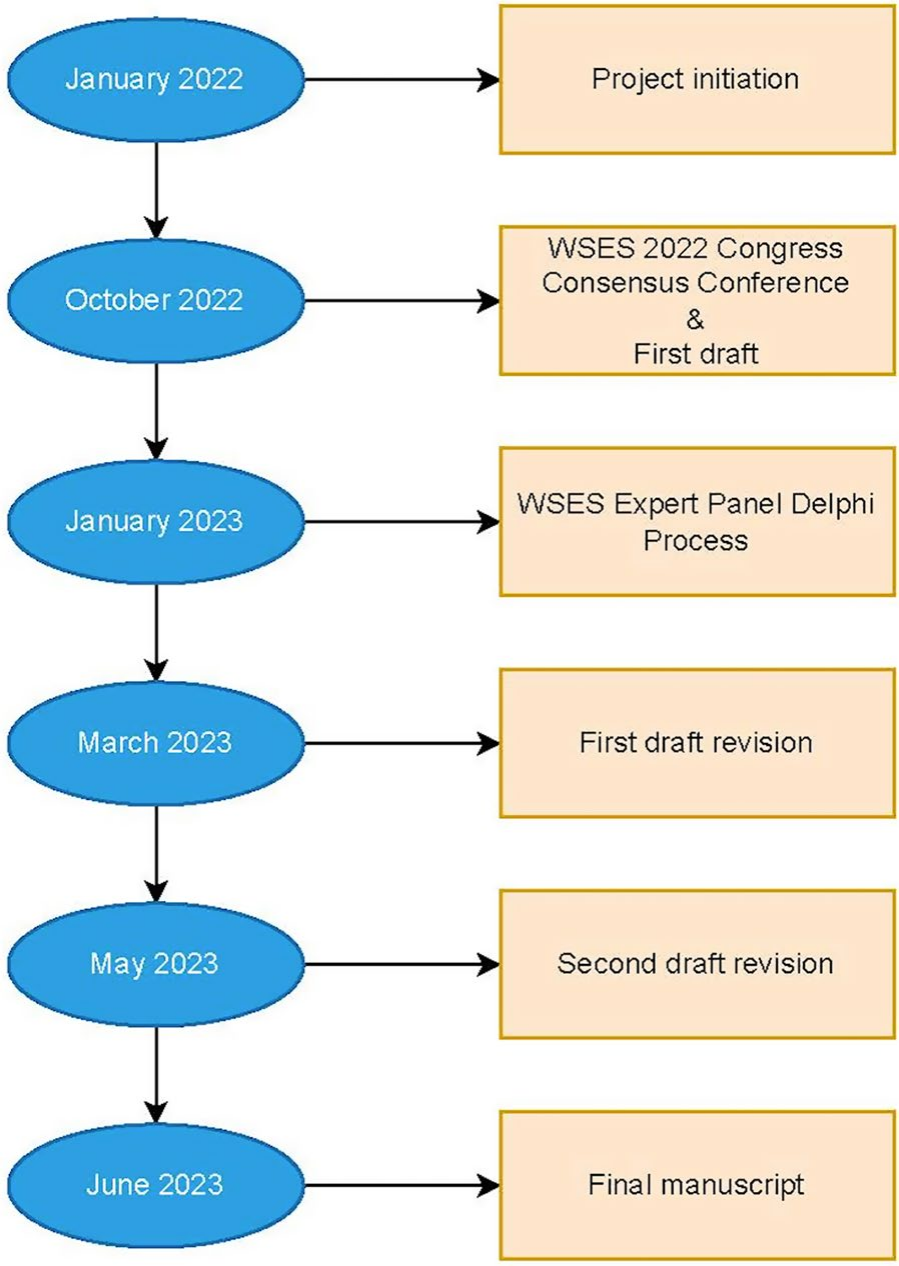
ECLAPTE project step-by-step process

With this objective in mind, in 2022, the World Society of Emergency Surgery (WSES) proposed a project called ECLAPTE (Effective Closure of LAParotomy in Emergency) to develop guidelines on the closure of emergency laparotomies following a previous survey among the WSES members. After a preliminary identification of the key questions, the evidence-based recommendations were drafted and reviewed by representatives for each section. During the 9th International WSES Congress in Perth, Western Australia, a Consensus Conference reviewed the guidelines in-depth prior to a Delphi process involving the WSES Board of Directors (Fig. 1). This manuscript summarizes the evidence to date, as well as the results of the Delphi and expert opinion.

## Methods

A systematic informatic search of the English-language literature was conducted by the ‘Pavia Organizational group’ in Pubmed, Scopus, and EMBASE web databanks. The databases were screened without time restrictions up to 1 July 2022 using the keywords ‘laparotomy,’ ‘closure,’ ‘midline incision,’ ‘emergency,’ ‘abdominal wall incision,’ ‘laparotomic incision,’ ‘closure technique,’ ‘incisional hernia,’ ‘wound dehiscence,’ ‘fascial dehiscence,’ and ‘wound complications’: results were combined with words AND/OR. No search restrictions were imposed; comprehensive published manuscripts of clinical trials, consensus conferences, comparative studies, guidelines, multicenter studies, systematic reviews, meta-analyses, large case series, original articles, and randomized clinical trials were considered.

A survey of WSES members guided the scope of topics for this guideline. Subsequently, representatives responsible from the Organizational Group identified six relevant sections to be investigated in depth and created a draft version of the guideline statements. The certainty of evidence and strength of recommendations were determined using the ‘Grading of Recommendations Assessment, Development, and Evaluation (GRADE) methodology’ [16–18]. Specifically, the GRADE system is an evidence-based tool that systematically evaluates the available literature and grades the Certainty of Evidence (CoE) as ‘High’/‘Moderate’/‘Low/‘Very low’ and the Strength of Recommendation (SoR) as either ‘Strong’ or ‘Weak’ (Table 1).

**Table 1:** GRADE system to evaluate the level of evidence and recommendation

Grade of recommendation	Clarity of risk/benefit	Quality of supporting evidence	Implications
1A Strong recommendation, high-quality evidence	Benefits clearly outweigh risk and burdens, or vice versa	RCTs without important limitations or overwhelming evidence from observational studies	Strong recommendation, applies to most patients in most circumstances without reservation
1B Strong recommendation, moderate-quality evidence	Benefits clearly outweigh risk and burdens, or vice versa	RCTs with important limitations (inconsistent results, methodological flaws, indirect analyses, or imprecise conclusions) or exceptionally strong evidence from observational studies	Strong recommendation, applies to most patients in most circumstances without reservation
1C Strong recommendation, low-quality, or very low-quality evidence	Benefits clearly outweigh risk and burdens, or vice versa	Observational studies or case series	Strong recommendation but subject to change when higher quality evidence becomes available
2A Weak recommendation, high-quality evidence	Benefits closely balanced with risks and burden	RCTs without important limitations or overwhelming evidence from observational studies	Weak recommendation, best action may differ depending on the patient, treatment circumstances, or social values
2B Weak recommendation, moderate-quality evidence	Benefits closely balanced with risks and burden	RCTs with important limitations (inconsistent results, methodological flaws, indirect, or imprecise) or exceptionally strong evidence from observational studies	Weak recommendation, best action may differ depending on the patient, treatment circumstances, or social values
2C Weak recommendation, low-quality or very low-quality evidence	Uncertainty in the estimates of benefits, risks, and burden; benefits, risk, and burden may be closely balanced	Observational studies or case series	Very weak recommendation; alternative treatments may be equally reasonable and merit consideration

During the 9th World Congress of the WSES held in Perth, Western Australia, in October 2022, each section and each question were discussed and voted upon by the audience—votes were either ‘YES/AGREE’ or ‘NO/DISAGREE.’ The poll was recorded using the Congress official mobile phone app, and the representatives of the Organizational group could immediately check the percentage of agreement. In case of disagreement or any comments, the statement was modified following the discussion. After the Congress poll, the Organizational group reviewed the guidelines according to the comments, and the revised version was voted online using a Delphi approach among the WSES Board of Directors and experts in the field. At the end of this step-by-step process, statements were approved with an agreement of ≥ 80%.

The method has already been employed in other guideline papers from WSES: this project was undertaken according to the usual methodology from the Society [19, 20].

The Organizational panel communicated via e-mail to prepare and revise the final guideline manuscript: valuable suggestion and comments from the Delphi process poll were integrated into the final document. The manuscript was successively reviewed by all contributors and ultimately revised as the present document. Statements are summarized in Table 2.

**Table 2:** Statements summary

Section/topic	Key questions	Statement
I. Does the abdominal wall incision in emergency surgery cases influence the incidence of incisional hernia, burst abdomen, or open abdomen?		I.1 When urgent access to the peritoneal cavity is required, we recommend midline laparotomy because it is faster and allows the best approach to the abdomen. When clinical circumstances allow, we suggest avoiding a midline incision for an alternative incision (2A)
		I.2 We recommend AGAINST midline incision as the extraction site when laparoscopic interventions are performed (1A)
1. What is the optimal technique to close a laparotomy incision?	1.1 Continuous suturing versus interrupted sutures	The current evidence does not suggest any difference in the incidence of incisional hernia or dehiscence between continuous or interrupted sutures for fascial closure. However, the time taken for fascial closure is less with continuous closure. Therefore, we suggest a continuous suture technique of the midline abdominal wall incision in emergency settings (2A)
	1.2 Closure versus non-closure of the peritoneum	We recommend AGAINST separate closure of the peritoneum during the abdominal wall closure of emergency laparotomy (1B)
	1.3 Mass closure versus single-layer closure	For closure of abdominal midline incision in emergency surgery, no difference between mass closure or layered closure was observed in terms of incisional hernia and wound complications: we suggest mass closure because it is faster than layered closure which might be highly important when emergency surgery is performed (2B)
	1.4 Suture length-to-wound length ratio (SL/WL)	We recommend a suture-to-wound length ratio (SL/WL) of at least 4:1 for continuous closure of midline abdominal wall incisions in emergency surgery (1B)
	1.5 Small bites technique versus large bites technique	We suggest the closure of the midline laparotomy with a ‘small bite’ technique to prevent incisional hernia and wound complications in emergency surgery cases although the evidence stems from elective surgery cases (2C)
2. What is the optimal suture material to close a laparotomy incision?	2.1 Non-absorbable versus absorbable suture	There is currently no evidence to suggest that absorbable or non-absorbable sutures are better in terms of incisional hernia or surgical site infections. Absorbable sutures may decrease pain; therefore, we suggest slowly absorbable sutures for the closure of emergency laparotomy (2C)
	2.2 Rapidly absorbable suture versus slowly absorbable suture	When using an absorbable suture for the closure of midline incisions in the emergency setting, we suggest choosing a slowly absorbable material (2A)
	2.3 Monofilament suture versus multifilament suture	We recommend a monofilament suture material (slowly absorbable monofilament suture) in the closure of midline laparotomies in the emergency setting as they may decrease the incidence of incisional hernia (1A)
	2.4 Sutures impregnated with antibiotics	We recommend an antimicrobial-coated suture for the fascial closure of abdominal laparotomy in cases of clean-, clean-contaminated, and contaminated fields when it is available in the emergency setting (1B)
3. Suture needles and retention suture	3.1 Is there a role for retention sutures when closing a laparotomy in emergency settings?	There is currently no high-quality evidence literature to suggest that retention sutures decrease the incidence of wound dehiscence in patients undergoing emergency laparotomies. The panel did not reach consensus as to whether retention sutures should be used routinely in laparotomy closures in the emergency setting *The panel did not reach an agreement of at least 80%, and consequently, this statement cannot be considered as an indication in the current guidelines*
	3.2 Is there any difference using blunt tapered needle or sharp needle in closing abdominal wall after emergency laparotomy?	There are very limited data about a blunt tapered or sharp needle in closing different layers of emergency laparotomies. Therefore, no recommendations can be made, and further studies are needed to clarify this concept
4. Perioperative care	4.1/4.2 Wound irrigation in emergency laparotomy closure	To decrease surgical site infection occurrence after emergency surgery, we suggest prophylactic wound irrigation in clean, clean-contaminated, and contaminated fields of the surgery. We recommend not to use antibiotic irrigation. (2C)
		Povidone–iodine wound irrigation has been associated with lower SSI rates, but recent data suggest that this consideration should be reconsidered. We recommend future prospective high-quality trials to clarify this point (2C)
	4.3 Subcutaneous drains in laparotomy incisions	There is currently no evidence supporting the routine use of subcutaneous drains. Therefore, we suggest AGAINST the routine use of subcutaneous drains after emergency laparotomy (2A)
	4.4/4.5 Leaving skin open after midline laparotomy—delayed laparotomy closure	There is currently no evidence to support or refute delayed laparotomy closure: because of the high risk of SSIs, we suggest surgeons should consider DCS of surgical wounds compared to primary closure in case of contaminated and dirty incisions with purulent contamination (2B)
		When delayed closure of surgical incision is performed, we recommend a revision between two and five days postoperatively (1B)
	4.6 Postoperative restriction of activity	No recommendation about postoperative physical restriction after open abdominal surgery can be made due to the lack of evidence, and further trials are necessary
	4.7/4.8 Negative pressure wound therapy for wound healing after emergency laparotomy	In patients undergoing primary closure after emergency laparotomy with high risk for surgical site infections, we recommend prophylactic incisional NPWT dressing on the closed skin (1A)
		No recommendation about a specific type of incisional NPWT dressing can be made due to the lack of evidence
5. Prophylactic mesh augmentation	5.1 Is prophylactic mesh augmentation beneficial for closure of laparotomies in emergency settings?	We suggest the use of prophylactic mesh augmentation in the closure of midline laparotomies in emergency settings to decrease the risk of incisional hernia (2B) *The panel did not reach an agreement of at least 80%, and consequently, this statement will not be considered as a recommendation in the current guidelines*
	5.2 Which type of patients should be considered for prophylactic mesh augmentation?	We suggest considering prophylactic mesh augmentation, particularly in patients with an increased risk of incisional hernia development (2B)
	5.3 Which type of mesh, which mesh position and which type of mesh fixation should be considered for prophylactic mesh augmentation?	In light of current evidence, for prophylactic mesh augmentation no specific type of mesh can be recommended. There is uncertainty about the type, position, or the type of fixation that should be used when prophylactic mesh augmentation is performed after emergency laparotomy. Evidence about mesh positioning is heterogeneous: onlay mesh position and retromuscular position are both recommended, even in emergency surgery, but future perspectives are needed to clarify the role of other types of meshes—absorbable and biological, for example, as well as other mesh placement positions (2C)
6. Trocar wounds for laparoscopic surgery and single-port surgery	6.1/6.2 Trocar size and type	Trocar-site hernia rates increase when trocars of 10 mm or larger are used and when trocars are introduced midline. We recommend using the smaller trocar size appropriate for the procedure and on an off-midline location when possible (2C)
		Trocar-site hernia may increase when bladed trocars are used. Surgeons may consider using non-bladed trocars when available (2C)
	6.3 Closure of trocar incision	We suggest closing the fascial defect caused by the trocar placement when trocars of 10 mm or of larger sizes are used (2C)
	6.4/6.5 Single incision laparoscopic surgery and incisional hernia	We recommend conventional laparoscopic procedures over single incision laparoscopic surgery (SILS) due to a higher risk of incisional hernia with the SILS technique (1B)
		When SILS is performed, surgeons might consider meticulous fascia closure to decrease the risk of incisional hernia formation (2C)

These guidelines should be considered as an adjunctive tool for decision making in a field in which no evidence was clear until now. Still, they are not a substitute for the surgeon’s clinical consideration. The Organizational group will update the considerations in case of significant changes based on new evidence.

## Results

### Introductory section

**I. Does the abdominal wall incision in emergency surgery cases influence the incidence of incisional hernia, burst abdomen, or open abdomen?**

**I.1 When urgent access to the peritoneal cavity is required, we recommend midline laparotomy because it is faster and allows the best approach to the abdomen. When clinical circumstances allow, we suggest avoiding a midline incision for an alternative incision (2A).**

$$\begin{aligned} & {\mathbf{\# }}\user2{CoE:A}{\mathbf{-}}{\varvec{High}}{\mathbf{/\# }}\user2{SoR:Weak} \\ & \left[ {{\text{Panel}}\;{\text{participants}}:{125}/{\text{Vote}}\;{\text{percentage}}:{1}00\% /{\mathbf{Agreement}}\;{\mathbf{percentage}}:{\mathbf{92}}\% } \right] \\ \end{aligned}$$

**I.2 We recommend AGAINST midline incision as the extraction site when laparoscopic interventions are performed (1A).**

$$\begin{aligned} & {\mathbf{\# }}\user2{CoE:A}{\mathbf{ - }}{\varvec{High}}{\mathbf{/\# }}\user2{SoR:Strong} \\ & \left[ {{\text{Panel}}\;{\text{participants}}:{125}/{\text{Vote}}\;{\text{percentage}}:{1}00\% /{\mathbf{Agreement}}\;{\mathbf{percentage}}:{\mathbf{87}}.{\mathbf{2}}\% } \right] \\ \end{aligned}$$

An appropriate incision is fundamental to performing any surgical operations, and the choice of incision in case of laparotomy depends on the anatomical site of interest, elective or emergency setting, and personal preference. Laparotomy incisions can be classified as follows:

- Midline—a vertical incision through the skin, subcutaneous tissue, linea alba, and peritoneum.
- Paramedian—the ‘conventional medial incision’ in which the rectus sheath and muscles are transected close to the linea alba and the ‘lateral incision’ in which rectus is transected near the lateral border.
- Transverse—a possible supraumbilical transverse incision for access to the upper abdomen or a ‘Pfannenstiel’ infraumbilical transverse incision for access to the lower abdomen.
- Oblique—a typical subcostal/Kocher incision or the McBurney incision.

In the literature, many randomized trials and systematic reviews with a high certainty evidence compared midline incisions to alternative incisions: the incisional hernia rate was significantly lower after non-midline incisions, for both transverse and oblique approaches [21–24]. In addition, a transverse incision appears to have less negative impact on pulmonary function, wound dehiscence, burst abdomen, and postoperative pain than a midline laparotomy [25, 26].

On the other hand, midline laparotomy is faster and allows the best access to all the organs of the peritoneal cavity: it is still the incision of choice in an emergency setting when a patient in a hemodynamically unstable condition needs to be explored [13, 15].

Therefore, our group recommends a different laparotomy incision from the classic midline approach when clinical circumstances allow: when preoperative imaging clearly identifies the site of pathology, a transverse incision can be used for emergency general surgery. For example, a transverse incision can be the best approach for advanced appendicitis and then could be extended medially to perform a right colectomy if necessary; similarly, a subcostal incision can be used for a complicated duodenal perforation. In addition, when emergency surgery is performed laparoscopically, we recommend avoiding midline incisions for the extraction site. However, the lack of evidence regarding the length and the location of these alternative incisions must be considered as a limitation.

### Section 1

**1. What is the optimal technique to close a laparotomy incision?**

**1.1 Continuous versus interrupted suture**

**The current evidence does not suggest any difference in the incidence of incisional hernia or dehiscence between continuous or interrupted sutures for fascial closure. However, the time taken for fascial closure is less with continuous closure. Therefore, we suggest a continuous suture technique of the midline abdominal wall incision in emergency settings (2A).**

$$\begin{gathered} {\mathbf{\# }}\user2{CoE:A}{\mathbf{-}}{\varvec{High}}{\mathbf{/\# }}\user2{SoR:Weak} \hfill \\ \left[ {{\text{Panel}}\;{\text{participants}}:\;{126}/{\text{Vote}}\;{\text{percentage}}:{99}.{2}\% /{\mathbf{Agreement}}\;{\mathbf{percentage}}:{\mathbf{92}}.{\mathbf{8}}\% } \right] \hfill \\ \end{gathered}$$

The literature search identified five large meta-analyses addressing the evidence on suture technique in terms of continuous versus interrupted methods [1, 14, 27–29]. Additionally, some well-designed randomized clinical trials were considered [30, 31]. Guidelines from the European Hernia Society—in the original 2015 version—recommended continuous closure of the midline abdominal wall, but the updated version in 2022 also considered more recent evidence and downgraded the certainty of evidence with a weak strength of recommendation due to the inconsistency of the results [13, 15]. Most of the evidence related to elective surgery, and clear data in the emergency setting are lacking. Therefore, in line with the evidence from Peponis et al. randomized clinical trial, we infer that there is no significant difference between continuous or interrupted sutures in the closure of abdominal wall incision, but the continuous technique might be preferred based on the elective surgery evidence because it is faster [1, 14, 27–32].

**1.2 Closure versus non-closure of the peritoneum**

**We recommend AGAINST separate closure of the peritoneum during the abdominal wall closure of emergency laparotomy (1B).**

$$\begin{aligned} & {\mathbf{\# }}\user2{CoE:B}{\mathbf{ - }}{\varvec{Moderate}}{\mathbf{/\# }}\user2{SoR:Strong} \\ & \left[ {{\text{Panel}}\;{\text{participants}}:{126}/{\text{Vote}}\;{\text{percentage}}:{98}.{4}\% /{\mathbf{Agreement}}\;{\mathbf{percentage}}:{\mathbf{87}}.{\mathbf{1}}\% } \right] \\ \end{aligned}$$

Regarding the closure of the peritoneal layer in a midline laparotomy, the Cochrane Systematic review by Gurusamy et al. [33] concluded that there is no short-term or long-term benefit in peritoneal closure. This review included five randomized clinical trials (RCTs): inclusion criteria in the studies were heterogeneous—the type of incision, elective, or emergency setting—but the studies concluded that closure of the peritoneum is unnecessary [34–38]. There is considerable uncertainty in the benefits or harms of the single-layered closure of the peritoneum as a separate layer, but this procedure is likely to increase operating time in emergency surgery.

Closure of the peritoneum involves additional operating time and suture material, and no benefit is apparent from closure of peritoneum. Therefore, it does not seem necessary after a midline laparotomy, even in an emergency setting.

**1.3 Mass closure versus layered closure**

**For closure of abdominal midline incision in emergency surgery, no difference between mass closure or layered closure was observed in terms of incisional hernia and wound complications: we suggest mass closure because it is faster than layered closure which might be highly important when emergency surgery is performed (2B).**

$$\begin{aligned} & {\mathbf{\# }}\user2{CoE:B}{\mathbf{ - }}{\varvec{Moderate}}{\mathbf{/\# }}{\varvec{SoR}}{\mathbf{:}}{\varvec{Weak}} \\ & \left[ {{\text{Panel}}\;{\text{participants}}:{125}/{\text{Vote}}\;{\text{percentage}}:{1}00\% /{\mathbf{Agreement}}\;{\mathbf{percentage}}:{\mathbf{86}}.{\mathbf{4}}\% } \right] \\ \end{aligned}$$

We used the EHS 2015 guidelines [13] definition of ‘mass closure’ versus ‘layered closure.’

Definitions proposed by Muysoms et al. (EHS 2015 guidelines) were:

- *Mass closure* The midline incision is closed with a suture bite including all layers of the abdominal wall except the skin. With this approach, the suture includes the fascia layers, peritoneum (which may or may not be included), and superficial layers in a single bite. By definition, mass closure is a single-layer closure technique.
- *Layered closure* The incision is closed with more than one separate layer of fascial closure. Specifically, if the incision is midline, there is only one layer of fascia. If the incision is paramedian, then there are two layers of rectus sheet above the arcuate line. With this approach, the peritoneal surface is normally closed separately as a different layer in the suture, and the same is done for the subcutaneous layer.

The following studies by Patel et al. and van Rooijen et al. in a systematic review and meta-analysis in 2018 analyzed the different impact of mass versus layered closure techniques in both elective and emergency settings. No difference was noted in terms of incisional hernia or wound complications, considering RCTs of moderate/low/very low certainty of evidence [1, 29, 39].

Therefore, we concluded that mass closure should be preferred because it is faster and no additional complications have been demonstrated, but the certainty of evidence is low due to the lack of specific data in emergency surgery and the low certainty of evidence from previous studies.

**1.4 Suture Length-to-Wound Length ratio (SL/WL)**

**We recommend a suture-to-wound length ratio (SL/WL) of at least 4:1 for continuous closure of midline abdominal wall incisions in emergency surgery (1B).**

$$\begin{aligned} & {\mathbf{\# }}\user2{CoE:B}{\mathbf{ - }}{\varvec{Moderate}}/{\mathbf{\# }}{\varvec{SoR}}{\mathbf{:}}{\varvec{Strong}} \\ & \left[ {{\text{Panel}}\;{\text{participants}}:{126}/{\text{Vote}}\;{\text{percentage}}:{1}00\% /{\mathbf{Agreement}}\;{\mathbf{percentage}}:{\mathbf{98}}.{\mathbf{4}}\% } \right] \\ \end{aligned}$$

The suture technique investigated through the suture length-to-wound length ratio is of crucial importance to avoid the development of incisional hernia and wound complications. The beneficial effect of a high suture length (SL)/wound length (WL) ratio has already been demonstrated, and previous guidelines consider a critical value a ratio of 4:1 or more (Jenkins Rule) [13, 15, 40–43].

Recently, data from RCTs were summarized in both elective and emergency surgeries after vascular operations: abdominal closure with a suture-to-wound length ratio of more than 4:1 compared with less than 4:1 significantly reduces the risk of incisional hernia and other wound complications [44–46].

Therefore, a SL/WL ratio of 4:1 or higher reduces the risk of incisional hernia and wound complications. It is recommended to document and ascertain this ratio at every wound closure.

**1.5 ‘Small bite’ technique versus ‘large bite’ technique**

**We suggest the closure of the midline laparotomy with a ‘small bite’ technique to prevent incisional hernia and wound complications in emergency surgery cases although the evidence stems from elective surgery cases (2C).**

$$\begin{aligned} & {\mathbf{\# }}{\varvec{CoE}}{\mathbf{:}}{\varvec{C}}{-}{\varvec{Low}}/{\mathbf{\# }}{\varvec{SoR}}{\mathbf{:}}{\varvec{Weak}} \\ & \left[ {{\text{Panel}}\;{\text{participants}}:{126}/{\text{Vote}}\;{\text{percentage}}:{1}00\% /{\mathbf{Agreement}}\;{\mathbf{percentage}}:{\mathbf{88}}.{\mathbf{8}}\% } \right] \\ \end{aligned}$$

The ‘small bite’ technique in the closure of midline laparotomy consists of a tissue stitch of approximately 5 mm from the median wound edges and a distance of approximately 5 mm from the other stitch allowing surgeons to include only the aponeurosis and to ensure adequate distribution of tension on the edge of the incision [47]. The ‘large bite’ technique consists of a distance from the wound edge and between stitches of more than 10 mm.

The positive effects of small stitches on wound healing have been widely expressed: aponeurosis has limited possibilities for regeneration and cannot bridge over a large defect. With a large stitch, not only aponeurosis tissue is included, but also fat and muscle. In combination with increased intra-abdominal pressure, soft tissue can be compressed and damaged. This can result in slackening and separation of wound edges, tissue devitalization, and infection. A separation of wound edges of more than 10/12 mm during the first postoperative period has been strongly associated with the development of an incisional hernia.

Large RCTs of acceptable quality, systematic reviews, and previous guidelines have investigated outcomes from the closure techniques, showing that incisional hernias and wound complications are significantly lower with the ‘small bite’ suture technique [13, 15, 48, 49]. However, all the studies were in the elective setting. The only manuscript looking specifically at this comparison in the emergency setting was the one by Peponis et al. [31]. Therefore, we suggest using the ‘small bite’ technique with low certainty of evidence also in cases of midline emergency laparotomy, but future perspectives studies on this topic are necessary to prove the effectiveness of this technique.

### Section 2

**2. What is the optimal suture material to close a laparotomy incision?**

**2.1 Non-absorbable versus absorbable suture**

**There is currently no evidence to suggest that absorbable or non-absorbable sutures are better in terms of incisional hernia or surgical site infections. Absorbable sutures may decrease pain; therefore, we suggest slowly absorbable sutures for the closure of emergency laparotomy (2C).**

$$\begin{aligned} & {\mathbf{\# }}\user2{CoE:C} - {\varvec{Low}}/{\mathbf{\# }}\user2{SoR:Weak} \\ & \left[ {{\text{Panel}}\;{\text{participants}}: {125}/{\text{Vote}}\;{\text{percentage}}:{1}00\% /{\mathbf{Agreement}}\;{\mathbf{percentage}}:{\mathbf{90}}.{\mathbf{4}}\% } \right] \\ \end{aligned}$$

There are many RCTs of high and moderate certainty evidence and even some previous systematic reviews and meta-analyses which investigated incisional hernia rates and other wound complications for different suture materials [14, 29, 32, 50–52]. A possible bias looking at these trials could be a combination of different suture techniques. Nevertheless, all high-level evidence considered a laparotomy incision closure through a continuous running suture. Taking into account these considerations, evidence failed to identify a significant superiority of one suture material over the other to reduce incisional hernia rate after a midline laparotomy [13, 15]. Van’t Riet et al. systematic review, Naz et al. RCT, and mainly Patel et al. Cochrane review highlighted less wound pain and surgical site infections in the absorbable suture group compared to the non-absorbable, but they agree that there is no clear evidence for all the other outcomes [1, 27, 53]. Most of the studies we considered include both elective and emergency settings.

Therefore, we conclude that there is no clear evidence for a recommendation about suture material, but some evidence about secondary outcomes suggest that non-absorbable suture may be avoided after emergency midline laparotomy.

**2.2 Rapidly absorbable suture versus slowly absorbable suture**

**When using an absorbable suture for the closure of midline incisions in the emergency setting, we suggest choosing a slowly absorbable material (2A).**

$$\begin{aligned} & {\mathbf{\# }}\user2{CoE:A}{\mathbf{ - }}{\varvec{High}}{\mathbf{/\# }}\user2{SoR:Weak} \\ & \left[ {{\text{Panel}}\;{\text{participants}}:{126}/{\text{Vote}}\;{\text{percentage}}:{1}00\% /{\mathbf{Agreement}}\,{\mathbf{percentage}}:{\mathbf{99}}.{\mathbf{2}}\% } \right] \\ \end{aligned}$$

Randomized clinical trials and numerous systematic reviews reported a lower incisional hernia rate when closure of the midline incision is performed with a slowly absorbable suture, in both elective and emergency settings [27, 28, 30, 32, 52, 54–56]. Accordingly, Muysoms et al. (EHS 2015 guidelines) are not recommending the use of rapidly absorbable sutures—with a focus on the specific area of elective surgery—[13]. Most recent data do not confirm strong evidence supporting the implementation of slowly absorbable sutures: a trend of fewer incisional hernia and wound complications is confirmed but without statistical significance [14, 15, 29].

So, we recommend, based on the high and moderate certainty of evidence, a slowly absorbable suture for the closure of midline emergency laparotomy.

**2.3 Monofilament suture versus multifilament suture**

**We recommend a monofilament suture material (slowly absorbable monofilament suture) in the closure of midline laparotomies in the emergency setting as they may decrease the incidence of incisional hernia (1A).**

$$\begin{aligned} & {\mathbf{\# }}\user2{CoE:A}{\mathbf{-}}{\varvec{High}}{\mathbf{/\# }}{\varvec{SoR}}{\mathbf{:}}{\varvec{Strong}} \\ & \left[ {{\text{Panel}}\;{\text{participants}}:{126}/{\text{Vote}}\;{\text{percentage}}:{1}00\% /{\mathbf{Agreement}}\;{\mathbf{percentage}}:{\mathbf{96}}.{\mathbf{8}}\% } \right] \\ \end{aligned}$$

Our literature research found evidence, suggesting that monofilament sutures are associated with a significantly lower risk of incisional hernia than multifilament sutures in both elective and emergency settings [1, 13, 15, 29]. On the other hand, no evidence specifically about wound complications—wound infections, wound dehiscence, wound sinus, and fistula formation—emerged from previous high/moderate certainty of evidence.

Therefore, according to our previous statements, if a slowly absorbable suture is used, a monofilament material is the only possible choice.

Because of the significant amount of data supporting the lower incidence of incisional hernia in emergency surgical settings with monofilament sutures, we have made a strong recommendation.

**2.4 Antimicrobial-coated sutures**

**We recommend an antimicrobial-coated suture for the fascial closure of abdominal laparotomy in cases of clean-, clean-contaminated, and contaminated fields when it is available in the emergency setting (1B).**

$$\begin{aligned} & {\mathbf{\# }}{\varvec{CoE}}{\mathbf{:}}{\varvec{B}}{\mathbf{-}}{\varvec{Moderate}}/{\mathbf{\# }}{\varvec{SoR}}{\mathbf{:}}{\varvec{Strong}} \\ & \left[ {{\text{Panelparticipants}}:{126}/{\text{Vote}}\;{\text{percentage}}:{1}00\% /{\mathbf{Agreement}}\;{\mathbf{percentage}}:{\mathbf{80}}.{\mathbf{1}}\% } \right] \\ \end{aligned}$$

Surgical site infections (SSIs) represent a common and serious complication of all surgical procedures, but it is even of greater concern in emergency surgery cases. Antimicrobial-coated sutures—typically triclosan-impregnated—have recently become a topic that generates considerable discussion, and is a well-known tool for preventing SSI, but they remain controversial due to elevated costs, worldwide availability, and the uncertainty in significant benefit for their use [55, 57–67].

Recently, high-quality RCTs in emergency settings and systematic reviews from Ahmed et al. and Uchino et al. have reported a significantly lower rate of surgical site infections when antibiotic-impregnated sutures are used in the closure of laparotomy in clean-, clean-contaminated, and contaminated fields [68–70].

Accordingly, our group is recommending antibiotic-coated suture in the emergency setting when it is available.

### Section 3

**3. Retention suture and suture needles**

**3.1 Is there a role for retention suture when closing a laparotomy in emergency setting?**

**There is currently no high-quality evidence literature to suggest that retention sutures decrease the incidence of wound dehiscence in patients undergoing emergency laparotomies. The panel did not reach consensus as to whether retention sutures should be used routinely in laparotomy closures in the emergency setting.**

$$\begin{aligned} & \left[ {{\text{Panel}}\;{\text{participants}}:{125}/{\text{Vote}}\;{\text{percentage}}:{99}.{2}\% /{\mathbf{Agreement}}\;{\mathbf{percentage}}:{\mathbf{68}}\% \;{\mathbf{on}}} \right. \\ & \left. {{\mathbf{avoiding}}\;{\mathbf{the}}\,{\mathbf{use}}\,{\mathbf{of}}\;{\mathbf{retention}}\;{\mathbf{sutures}}\;{\mathbf{routinely}}} \right] \\ \end{aligned}$$

***The panel did not reach an agreement of at least 80%, and consequently, this statement cannot be considered as an indication in the current guidelines.***

No systematic review was found regarding the implementation of retention sutures in the closure of laparotomy, in the elective or emergency surgery setting. Guidelines and indications for the prophylactic use of retention sutures are lacking and not clear. Nevertheless, some evidence supports the use of this technique in the case of [71, 72]:

- Patients with increased tension in the incision;
- Patients with preoperative severe malnutrition;
- Patients who are immunocompromised;
- Patients with previous fascial defects;
- Patients with massive abdominal contamination.

‘Retention suture technique’ includes a suture outside from the primary incision site line through all layers of the abdominal wall, including the skin, with a large-bore non-absorbable suture material. Various tools are available to alleviate the tension of the retention suture on the skin for patients’ comfort. The effect is to reduce the tension on the primary suture line.

In the studies which were screened by our group, the target of the trials was always patients with an emergency indication for midline incisions or elective surgery in patients with high-risk factors for wound complications. Some randomized clinical trials with moderate certainty of evidence were considered. The principal outcomes in these studies were heterogeneous, but surgical infections and wound dehiscence were typically investigated. In addition, follow-up was too short to identify any major laparotomy complications, even in oncological patients [72–79].

Anyhow, the evidence considered seems to suggest a lower incidence of wound dehiscence in the retention suture group. On the other hand, there is higher postoperative pain in the group of patients treated with retention sutures. Accordingly, retention sutures could be considered as a possible addition to suture closure of emergency laparotomy only in case of patients with very high-risk conditions for incisional hernia and wound dehiscence.

**3.2 Is there any difference between using a blunt tapered needle or a sharp needle in closing the abdominal wall after an emergency laparotomy?**

**There are very limited data about a blunt tapered or sharp needle in closing different layers of emergency laparotomies. Therefore, no recommendations can be made, and further studies are needed to clarify this concept.**

$${\mathbf{\# }}{\varvec{CoE}}{\mathbf{:}}{\varvec{D}}{-}{\varvec{Very}}\;{\varvec{low}}$$

***No voting was requested for this statement as there were no recommendations included in this statement.***

Only one randomized clinical trial comparing blunt tapered and the standard sharp needle in elective and emergency general surgery has been published [80]. This trial included 200 patients, and the main outcome was the surgical team safety in terms of the number of procedures with one or more glove perforations. The secondary outcome was the number of procedures with omentum or bowel puncture comparing the use of the blunt tapered or sharp needle. No data about surgical outcomes, such as incisional hernia, fascial dehiscence, wound complications, or postoperative pain were reported. On the other hand, additional evidence comes from gynecological studies: only in the RCT published by Stafford wound infections were the main outcome [81, 82]. Therefore, no recommendations can be given on the use of a different type of needle, but we can recommend the use of blunt tapered needles as an important tool in decreasing the number of incidental glove and visceral perforation.

### Section 4

**4. Perioperative care**

**4.1/4.2 Wound irrigation in emergency laparotomy closure**

**4.1 To decrease surgical site infection occurrence after emergency surgery, we suggest prophylactic wound irrigation in clean, clean-contaminated, and contaminated fields of the surgery. We recommend not to use antibiotic irrigation. (2C).**

$$\begin{aligned} & {\mathbf{\# }}{\varvec{CoE}}{\mathbf{:}}{\varvec{C}}{-}{\varvec{Low}}{\mathbf{/\# }}{\varvec{SoR}}{\mathbf{:}}{\varvec{Weak}} \\ & \left[ {{\text{Panel}}\;{\text{participants}}:{122}/{\text{Vote}}\,{\text{percentage}}:{99}.{1}\% /{\mathbf{Agreement}}\;{\mathbf{percentage}}:{\mathbf{90}}.{\mathbf{0}}\% } \right] \\ \end{aligned}$$

**4.2 Povidone–iodine wound irrigation has been associated with lower SSI rates, but recent data suggest that this consideration should be reconsidered. We recommend future prospective high-quality trials to clarify this point (2C).**

$$\begin{aligned} & {\mathbf{\# }}{\varvec{CoE}}{\mathbf{:}}{\varvec{C}}{\mathbf{-}}{\varvec{Low}}{\mathbf{/\# }}{\varvec{SoR}}{\mathbf{:}}{\varvec{Weak}} \\ & \left[ {{\text{Panel}}\;{\text{participants}}:{121}/{\text{Vote}}\;{\text{percentage}}:{99}.{1}\% /{\mathbf{Agreement}}\;{\mathbf{percentage}}:{\mathbf{93}}.{\mathbf{3}}\% } \right] \\ \end{aligned}$$

Surgical site infections (SSIs) are one of the most common hospital-acquired infections. SSIs are a preventable complication, responsible for substantial costs to health services that can result in poorer patient outcomes, increased mortality, morbidity, and reoperation rates. While the cause of SSIs is multifactorial, wounds can be classified by their level of contamination as suggested by the Centers for Disease Control and Prevention (CDC):

- Class I/Clean
- Class II/Clean—Contaminated
- Class III/Contaminated
- Class IV/Dirty—Infected

Based on the included trial evidence, there is currently no clear difference in the incidence of SSIs between patients treated with irrigation and without irrigation, with low-grade certainty of less incidence of SSIs when irrigation is performed [83, 84].

In addition, there is not a clear indication about the type of surgical wound irrigation that could be more beneficial in the setting of emergency laparotomies: evidence from Norman et al. Cochrane systematic review and meta-analysis support the implementation of antibacterial irrigation compared with non-antibacterial irrigation, whereas de Jonge et al. systematic review and meta-analysis show that antibiotic irrigation does not offer a benefit, contribute to antimicrobial resistance and prophylactic incisional wound irrigation to prevent SSI rates with an aqueous povidone–iodine solution should be considered [85, 86]. According to this evidence, povidone–iodine wound irrigation is associated with lower SSI rates compared to saline-only wound irrigation: this consideration is not specific to emergency surgery but is based on elective surgery settings [87]. Recent data from Chinese and Japanese RCTs query the povidone–iodine wound irrigation superiority in the prevention of SSIs, suggesting that the current recommendation should be reconsidered in light of future prospective high-quality trials [88, 89].

Therefore, our group suggest the use of wound irrigation, but future evidence on the best irrigation technique is necessary.

**4.3 Subcutaneous drains in emergency laparotomy incisions**

**There is currently no evidence supporting the routine use of subcutaneous drains. Therefore, we suggest AGAINST the routine use of subcutaneous drains after emergency laparotomy (2A).**

$$\begin{aligned} & {\mathbf{\# }}{\varvec{CoE}}{\mathbf{:}}{\varvec{A}}{\mathbf{-}}{\varvec{High}}{\mathbf{/\# }}{\varvec{SoR}}{\mathbf{:}}{\varvec{Weak}} \\ & \left[ {{\text{Panel}}\;{\text{participants}}:{124}/{\text{Vote}}\,{\text{percentage}}:{99}.{1}\% /{\mathbf{Agreement}}\;{\mathbf{percentage}}:{\mathbf{95}}.{\mathbf{9}}\% } \right] \\ \end{aligned}$$

Surgical site infection (SSI) is considered a postoperative complication after surgery that increases patient morbidity and mortality rates. Some authors suggest the use of a subcutaneous drain to prevent wound infection, but high-quality-of-evidence systematic reviews and meta-analyses demonstrate that the routine placement of a subcutaneous drain during the closure of abdominal wall incision does not confer any advantage in preventing postoperative wound infection [13].

Coletta et al. systematic review and meta-analysis in 2019 suggest that subcutaneous drains should not be used routinely, as it does not confer any advantage in preventing postoperative wound infection, but this does not exclude that there might be a benefit in a specific risk group of patients [90]. We found a recent RCT of moderate level of evidence by Harish et al., which stated that subcutaneous suction drains have been shown to reduce SSIs in a large number of patients [91]. One hundred patients were studied in this trial; however, the inconsistency in the results and publication bias means that our group cannot make a strong recommendation [91]. It is important to obliterate any dead space by using quilting sutures.

Therefore, our group is not recommending the implementation of drains in perioperative treatment of midline laparotomy incision until evidence from future trials of low risk of bias.

**4.4/4.5 Delayed Closure of the Skin (DCS)—Leaving skin open after midline laparotomy.**

**4.4 There is currently no evidence to support or refute delayed laparotomy closure: because of the high risk of SSIs, we suggest surgeons should consider DCS of surgical wounds compared to primary closure in case of contaminated and dirty incisions with purulent contamination (2B).**

$$\begin{aligned} & {\mathbf{\# }}\user2{CoE:B}{\mathbf{-}}{\varvec{Moderate}}/{\mathbf{\# }}{\varvec{SoR}}{\mathbf{:}}{\varvec{Weak}} \\ & \left[ {{\text{Panel}}\;{\text{participants}}:{124}/{\text{Vote}}\;{\text{percentage}}:{99}.{1}\% /{\mathbf{Agreement}}\;{\mathbf{percentage}}:\;{\mathbf{83}}.{\mathbf{5}}\% } \right] \\ \end{aligned}$$

**4.5 When delayed closure of surgical incision is performed, we recommend a revision between two and five days postoperatively (1B).**

$$\begin{aligned} & {\mathbf{\# }}{\varvec{CoE}}{\mathbf{:}}{\varvec{B}}{\mathbf{-}}{\varvec{Moderate}}/{\mathbf{\# }}{\varvec{SoR}}{\mathbf{:}}{\varvec{Strong}} \\ & \left[ {{\text{Panel}}\;{\text{participants}}:{95}/{\text{Vote}}\;{\text{percentage}}:{98}.{9}\% /{\mathbf{Agreement}}\;{\mathbf{percentage}}:{\mathbf{96}}.{\mathbf{8}}\% } \right] \\ \end{aligned}$$

Surgical site infections (SSI) following abdominal surgery are common and confer significant morbidity. Therefore, there is a strong interest in reducing the rate of SSI globally.

In addition to the practical tools for the proper closure of laparotomy incisions, some procedures and techniques for skin closure have been investigated to achieve a lower rate of SSI. Delayed primary closure (DPC) and primary closure (PC) are the most commonly used methods: DPC can be used when contaminated and dirty wounds with purulent contamination are created and it consists in leaving the skin open to allow soft tissue drains, PC is the classical direct closure of all anatomical layers—skin included -. Currently, there is no consensus on the optimal method and no indication of the best clinical practice has been reported [92, 93].

Emergency surgery procedures are at significant risk of contamination due to the types of interventions that are performed daily. Therefore, we were able to find some high and moderate level-of-evidence randomized clinical trials comparing DPC and PC in our search. Based on evidence by Banghu et al., delayed skin closure seems to reduce SSI rates, but the trial had a high risk of bias, and random effect model showed no evidence of difference [94]. There is no strong evidence to support one method over another.

Finally, we found concordant data on a surgical second look with closure, if no wound complications are noticed, between the second and fifth postoperative day [92–94].

**4.6 Postoperative restriction of activity**

**No recommendation about postoperative physical restriction after open abdominal surgery can be made due to the lack of evidence, and further trials are necessary.**

***No voting was requested for this statement because no specific recommendation is being made.***

There are very limited data on the optimal time of physical restriction of activity after open laparotomy surgery. In the literature research, only one systematic review was found surgeons suggest a variable period of convalescence and physical inactivity to reduce the risk of incisional hernia, but this period usually ranged from 1 week and 3 months for different types of approaches and procedures [95].

On the other hand, as stated by Enhanced Recovery After Surgery (ERAS) Society Recommendations in elective colorectal surgery, early mobilization after abdominal surgery is widely regarded as an important component of perioperative care. Prolonged immobilization is associated with various adverse effects and patients should therefore be encouraged to increase a rapid return to movements and walks after surgery. No data are available about the timing of early mobilization in terms of postoperative days [96].

Therefore, no recommendations can be given on restriction of activity after open abdominal surgery and randomized controlled trials are necessary to state a safe period of recovery.

**4.7/4.8 Negative pressure wound therapy (NPWT) for wound healing after emergency laparotomy**

**4.7 In patients undergoing primary closure after emergency laparotomy with high risk for surgical site infections, we recommend prophylactic incisional NPWT dressing on the closed skin (1A).**

$$\begin{aligned} & {\mathbf{\# }}{\varvec{CoE}}{\mathbf{:}}{\varvec{A}}{-}{\varvec{High}}/{\mathbf{\# }}{\varvec{SoR}}{\mathbf{:}}{\varvec{Strong}} \\ & \left[ {{\text{Panel}}\;{\text{participants}}:{125}/{\text{Vote}}\;{\text{percentage}}:{1}00\% /{\mathbf{Agreement}}\;{\mathbf{percentage}}:{\mathbf{92}}.{\mathbf{8}}\% } \right] \\ \end{aligned}$$

**4.8 No recommendation about a specific type of incisional NPWT dressing can be made due to the lack of evidence.**

***No voting was requested for this statement as there was no recommendation.***

Patients undergoing emergency laparotomy—with or without bowel surgery—are particularly at risk for surgical site infections (SSI). Incisional negative pressure wound therapy (iNPWT) has been shown to reduce surgical site infections in the elective setting, but until recently data were limited to the emergency setting [15]. Some observational retrospective studies with propensity-matched analysis, systematic reviews and meta-analysis, and a Cochrane review have been published after 2019 aiming to assess the role of iNPWT in trauma and emergency surgery [97–100].

Data suggest that in a population at high risk of development of SSI, iNPWT resulted in a lower risk of wound infections. Patients undergoing emergency laparotomy for a gastrointestinal procedure and at high risk of developing SSI, seem to be the target population in which iNPWT has beneficial effects [101, 102].

No specific data about the type of iNPWT to be used (e.g., PICO, PREVENA, others) were identified; thus, our group could not make any specific recommendation about it.

### Section 5

**5. Prophylactic mesh augmentation**

**5.1 Is prophylactic mesh augmentation beneficial for the closure of laparotomies in emergency settings?**

*The original version of this statement*—*in light of the evidence from literature and after the open discussion at the 9th WSES International Congress in Perth, Western Australia*—*was submitted in a Delphi process to the WSES Board of Directors and experts: the panel did not reach an agreement of at least 80%, and consequently, this statement will not be considered as a recommendation in the current guidelines.*

*We report the original version of the statement with the agreed percentage from the panel polling*.

**We suggest the use of prophylactic mesh augmentation in the closure of midline laparotomies in emergency settings to decrease the risk of incisional hernia (2B).**

$$\begin{aligned} & {\mathbf{\# }}{\varvec{CoE}}{\mathbf{:}}{\varvec{B}}{-}{\varvec{Moderate}}{\mathbf{/\# }}{\varvec{SoR}}{\mathbf{:}}{\varvec{Weak}} \\ & \left[ {{\text{Panel}}\;{\text{participants}}:{121}/{\text{Vote}}\;{\text{percentage}}:{1}00\% /{\mathbf{Agreement}}\;{\mathbf{percentage}}:{\mathbf{67}}.{\mathbf{7}}\% } \right] \\ \end{aligned}$$

The current evidence on the efficacy of prophylactic mesh augmentation is overwhelming. Data highlight a significant reduction in the incisional hernia rate in elective settings, and trends suggest a significantly lower rate of incisional hernia in emergency laparotomies [13, 15, 103]. Prophylactic mesh augmentation after midline incision significantly impacts incisional hernia and does not predict an increased risk of postoperative complications [104–115]. However, a systematic review on the use of mesh in emergency surgery included only two small RCTs. The evidence does not suggest that the wound failure is lower in the mesh group [104]. Other observational studies are likely biased, and data must be regarded carefully, specifically about SSI rates. A large number of surgeons are still concerned about mesh reinforcement in cases of contaminated surgery.

Recent meta-analyses confirm robust evidence supporting the role of mesh as prophylactic augmentation in the closure of the abdominal wall after laparotomy: incisional hernia rate decreased significantly, but on the other hand, an increased trend in wound complications was identified. Consequently, we decided to downgrade our recommendation to a suggestion.

Anyhow, in the closure of midline laparotomy incisions in an emergency setting– specifically in the case of high-risk patients for fascial dehiscence—prophylactic mesh augmentation appears to be effective in preventing incisional hernia and safe for postoperative hospitalization.

**5.2 Which type of patients should be considered for prophylactic mesh augmentation?**

**We suggest considering prophylactic mesh augmentation, particularly in patients with an increased risk of incisional hernia development (2B).**

$$\begin{aligned} & & {\mathbf{\# }}{\varvec{CoE}}{\mathbf{:}}{\varvec{B}}{\mathbf{-}}{\varvec{Moderate}}/{\mathbf{\# }}{\varvec{SoR}}{\mathbf{:}}{\varvec{Weak}} \\ & \left[ {{\text{Panel}}\;{\text{participants}}:{113}/{\text{Vote}}\;{\text{percentage}}:{97}.{3}\% /{\mathbf{Agreement}}\;{\mathbf{percentage}}:{\mathbf{80}}\% } \right] \\ \end{aligned}$$

Risk factors for postoperative hernia development are already reported in the introduction section of this paper [11, 12].

**5.3 Which type of mesh, which mesh position, and which type of mesh fixation should be considered for prophylactic mesh augmentation?**

**In light of current evidence, for prophylactic mesh augmentation no specific type of mesh can be recommended.**

**There is uncertainty about the type, position, or the type of fixation that should be used when prophylactic mesh augmentation is performed after emergency laparotomy. Evidence about mesh positioning is heterogeneous: onlay mesh position and retromuscular position are both recommended, even in emergency surgery, but future perspectives are needed to clarify the role of other types of meshes—absorbable and biological, for example, as well as other mesh placement positions (2C).**

$$\begin{aligned} & {\mathbf{\# }}{\varvec{QoE}}{\mathbf{:}}{\varvec{C}}{\mathbf{-}}{\varvec{Low}}{\mathbf{/\# }}{\varvec{SoR}}{\mathbf{:}}{\varvec{Weak}} \\ & \left[ {{\text{Panel}}\;{\text{participants}}:{117}/{\text{Vote}}\;{\text{percentage}}:{1}00\% /{\mathbf{Agreement}}\;{\mathbf{percentage}}:{\mathbf{87}}.{\mathbf{1}}\% } \right] \\ \end{aligned}$$

No studies specifically compare the types of mesh used as prophylactic mesh augmentation. In terms of incisional hernia and postoperative surgical incision complications, in the series we analyzed, different types of mesh were analyzed: absorbable synthetic, non-absorbable synthetic, and biological meshes [116, 118]. Most guidelines and systematic reviews try to investigate any difference in the wound dehiscence rate after absorbable or non-absorbable mesh implantation, but clear data are lacking. None of the randomized clinical trials we considered highlight any significant differences in incisional hernia rates between different prophylactic mesh types. In addition, only a few randomized clinical trials specifically investigated prophylactic mesh types in the emergency setting and the occurrence of incisional hernias or postoperative complications. Accordingly, synthetic non-absorbable, absorbable, and biological meshes should be considered even in the emergency setting. Future studies are needed to clarify the most appropriate mesh position and fixation techniques.

Only a few randomized clinical trials compared different prophylactic mesh placements, without a high level of evidence in these analyses. Most studies investigated the role of mesh implantation in an onlay or retromuscular positions showing a significant reduction in the incidence of incisional hernias; however, a higher risk of wound complications has been reported in most series when compared to primary closure alone [117]. In the specific subgroup of cases treated in the emergency setting, prophylactic onlay or retromuscular mesh augmentation the evidence reported in the elective setting is confirmed [12]. Although a lower rate of incisional hernia was reported, there is a lack of evidence about long-term complications following the intraperitoneal prophylactic mesh positioning. In addition, there are several concerns about the use in contaminated fields and the increased risk of adhesive complications. No data were found about outcomes according to different techniques of mesh fixation.

### Section 6

**6. Trocar wounds for laparoscopic surgery and single-port surgery**

**6.1/6.2 Trocar size and type**

**6.1 Trocar-site hernia rates increase when trocars of 10 mm or larger are used and when trocars are introduced midline. We recommend using the smaller trocar size appropriate for the procedure and on an off-midline location when possible (2C).**

$$\begin{aligned} & {\mathbf{\# }}{\varvec{CoE}}{\mathbf{:}}{\varvec{C}}{\mathbf{-}}{\varvec{Low}}/{\mathbf{\# }}{\varvec{SoR}}{\mathbf{:}}{\varvec{Weak}} \\ & \left[ {{\text{Panel}}\;{\text{participants}}:{117}/{\text{Vote}}\;{\text{percentage}}:{1}00\% /{\mathbf{Agreement}}\;{\mathbf{percentage}}:{\mathbf{89}}.{\mathbf{7}}\% } \right] \\ \end{aligned}$$

**6.2 Trocar-site hernia may increase when bladed trocars are used. Surgeons may consider using non-bladed trocars when available (2C).**

$$\begin{aligned} & {\mathbf{\# }}{\varvec{CoE}}{\mathbf{:}}{\varvec{C}}{\mathbf{-}}{\varvec{Low}}/{\mathbf{\# }}{\varvec{SoR}}{\mathbf{:}}{\varvec{Weak}} \\ & \left[ {{\text{Panel}}\;{\text{participants}}:{117}/{\text{Vote}}\;{\text{percentage}}:{1}00\% /{\mathbf{Agreement}}\;{\mathbf{percentage}}:{\mathbf{89}}.{\mathbf{7}}\% } \right] \\ \end{aligned}$$

**6.3 Closure of trocar incision**

**We suggest closing the fascial defect caused by the trocar placement when trocars of 10 mm or of larger sizes are used (2C).**

$$\begin{aligned} & {\mathbf{\# }}{\varvec{CoE}}{\mathbf{:}}{\varvec{C}}{\mathbf{-}}{\varvec{Low}}{\mathbf{/\# }}{\varvec{SoR}}{\mathbf{:}}{\varvec{Weak}} \\ & \left[ {{\text{Panel}}\;{\text{participants}}:{116}/{\text{Vote}}\;{\text{percentage}}:{1}00\% /{\mathbf{Agreement}}\;{\mathbf{percentage}}:{\mathbf{93}}.{\mathbf{9}}\% } \right] \\ \end{aligned}$$

**6.4/6.5 Single incision laparoscopic surgery and incisional hernia**

**6.4 We recommend conventional laparoscopic procedures over single incision laparoscopic surgery (SILS) due to a higher risk of incisional hernia with the SILS technique (1B).**

$$\begin{aligned} & {\mathbf{\# }}{\varvec{CoE}}{\mathbf{:}}{\varvec{B}}{\mathbf{ - }}{\varvec{Moderate}}{\mathbf{/\# }}\user2{SoR:Strong} \\ & \left[ {{\text{Panel}}\;{\text{participants}}:{116}/{\text{Vote}}\;{\text{percentage}}:{1}00\% /{\mathbf{Agreement}}\;{\mathbf{percentage}}:{\mathbf{89}}.{\mathbf{6}}\% } \right] \\ \end{aligned}$$

**6.5 When SILS is performed, surgeons might consider meticulous fascia closure to decrease the risk of incisional hernia formation (2C).**

$$\begin{aligned} & {\mathbf{\# }}{\varvec{CoE}}{\mathbf{:}}{\varvec{C}}{\mathbf{ - }}{\varvec{Low}}{\mathbf{/\# }}{\varvec{SoR}}{\mathbf{:}}{\varvec{Weak}} \\ & \left[ {{\text{Panel}}\;{\text{participants}}:{116}/{\text{Vote}}\;{\text{percentage}}:{1}00\% /{\mathbf{Agreement}}\;{\mathbf{percentage}}:{\mathbf{99}}.{\mathbf{1}}\% } \right] \\ \end{aligned}$$

Trocar-site hernia (TSH) is a rare complication of laparoscopic surgery with a likely under-reported incidence of 0.1–1.0%. The literature on the topic is heterogeneous and typically reports data on elective and bariatric surgery cases. In our search, we identified only four systematic reviews including patients treated in the emergency setting and without any subgroup analysis; the quality of evidence was very low/low/moderate [119–121].

Regarding trocar size and location, there appears to be a higher risk of TSH when trocars of 10 mm or larger are used and when trocars are placed in the midline [122, 123]. In addition, clear evidence comparing bladed versus non-bladed trocars underlines a statistically significant lower incidence of TSH with non-bladed instruments.

Before the recent systematic review by Gutierrez et al., there was a consensus on the indication of fascial closure for trocar sites of 10 mm or more; in this recent paper, comparing fascial closure between 5 and 10 mm ports, no difference in TSH was reported, although leaving the fascia open may reduce operative time [124]. No specific data about the emergency setting is reported on this topic. Accordingly, we downgraded the strength of recommendation of our statement as further studies are necessary to clarify this issue.

Two systematic reviews of moderate certainty about single incision laparoscopic surgery (SILS) compared to traditional multiport laparoscopic surgery were published. The first reports on a variety of surgical procedures, and the second focuses only on laparoscopic cholecystectomy cases [125, 126]. Both studies showed an increased risk of incisional hernia after SILS compared to conventional laparoscopy. Therefore, we recommend conventional laparoscopy procedures instead of SILS. If SILS is performed, meticulous fascia closure is mandatory.

## Conclusions

Incisional hernias and postoperative complications (wound dehiscence, fascial dehiscence, and surgical site infections) represent frequent complications after midline laparotomy, which is still the best approach to the abdomen in case of emergency settings for trauma, intra-abdominal sepsis management, and other acute abdominal conditions. The estimated incidence of IHs following major abdominal surgery ranges from 2 to 40% across studies, considering both elective and emergency procedures [1, 2]. The previous version of international guidelines investigated the abdominal wall closure techniques exclusively in elective surgery but provided no specific recommendations about emergency surgery cases.

A panel of experts from the World Society of Emergency Surgery discussed a series of key questions in a double-step process, firstly during the 9th World Congress of the WSES and then through a Delphi questionnaire among the WSES Board of Directors. The ECLAPTE project defined the optimal technique to close a laparotomy incision performed in the emergency setting, the optimal suture material, the role of retention sutures, and provided some advice about perioperative care. Our review focused on innovative and modern aspects of acute care surgery and trauma care. We examined the role of prophylactic mesh augmentation and provided suggestions about laparoscopic surgery when performed.

Moreover, areas for important future research were identified. Wound irrigation solutions, period of restriction from physical activity, the type and position of prophylactic mesh are relevant topics for future investigations. In addition, a significant point missing in literature, notorious neglected IHs site, is drain site incision: future perspectives are needed to clarify also the best closure technique for this incision.

Finally, the WSES advocates the adoption of these guidelines as a safe and evidence-based common approach in the emergency setting, but at the same time it encourages the development of local pathways based on the available evidence and resources.

## Data Availability

Not applicable.

## Abbreviations

IH: Incisional hernia
WSES: World Society of Emergency Surgery
EHS: European Hernia Society
CoE: Certainty of evidence
SoR: Strength of recommendation
RCTs: Randomized clinical trials
SL: Suture length
WL: Wound length
SSIs: Surgical site infections
CDC: Center for Disease Control and Prevention
DCS: Delayed laparotomy closure
PC: Primary closure
ERAS: Enhanced Recovery After Surgery
iNPWT: Incisional negative pressure wound therapy
TSH: Trocar-site hernia
SILS: Single Laparoscopic Surgery
