# Correction: ECLAPTE: *Effective Closure of LAParoTomy in Emergency*—2023 World Society of Emergency Surgery guidelines for the closure of laparotomy in emergency settings

**DOI:** 10.1186/s13017-023-00522-7

**Published:** 2023-11-27

**Authors:** Simone Frassini, Lorenzo Cobianchi, Paola Fugazzola, Walter L. Biffl, Federico Coccolini, Dimitrios Damaskos, Ernest E. Moore, Yoram Kluger, Marco Ceresoli, Raul Coimbra, Justin Davies, Andrew Kirkpatrick, Isidoro Di Carlo, Timothy C. Hardcastle, Arda Isik, Massimo Chiarugi, Kurinchi Gurusamy, Ronald V. Maier, Helmut A. Segovia Lohse, Hans Jeekel, Marja A. Boermeester, Fikri Abu-Zidan, Kenji Inaba, Dieter G. Weber, Goran Augustin, Luigi Bonavina, George Velmahos, Massimo Sartelli, Salomone Di Saverio, Richard P. G. Ten Broek, Stefano Granieri, Francesca Dal Mas, Camilla Nikita Farè, Jacopo Peverada, Simone Zanghì, Jacopo Viganò, Matteo Tomasoni, Tommaso Dominioni, Enrico Cicuttin, Andreas Hecker, Giovanni D. Tebala, Joseph M. Galante, Imtiaz Wani, Vladimir Khokha, Michael Sugrue, Thomas M. Scalea, Edward Tan, Mark A. Malangoni, Nikolaos Pararas, Mauro Podda, Belinda De Simone, Rao Ivatury, Yunfeng Cui, Jeffry Kashuk, Andrew Peitzman, Fernando Kim, Emmanouil Pikoulis, Gabriele Sganga, Osvaldo Chiara, Michael D. Kelly, Ingo Marzi, Edoardo Picetti, Vanni Agnoletti, Nicola De’Angelis, Giampiero Campanelli, Marc de Moya, Andrey Litvin, Aleix Martínez-Pérez, Ibrahima Sall, Sandro Rizoli, Gia Tomadze, Boris Sakakushev, Philip F. Stahel, Ian Civil, Vishal Shelat, David Costa, Alain Chichom-Mefire, Rifat Latifi, Mircea Chirica, Francesco Amico, Amyn Pardhan, Vidya Seenarain, Nikitha Boyapati, Basil Hatz, Travis Ackermann, Sandun Abeyasundara, Linda Fenton, Frank Plani, Rohit Sarvepalli, Omid Rouhbakhshfar, Pamela Caleo, Victor Ho-Ching Yau, Kristenne Clement, Erasmia Christou, Ana María González Castillo, Preet K. S. Gosal, Sunder Balasubramaniam, Jeremy Hsu, Kamon Banphawatanarak, Michele Pisano, Adriana Toro, Altomare Michele, Stefano P. B. Cioffi, Andrea Spota, Fausto Catena, Luca Ansaloni

**Affiliations:** 1 University of Pavia, Corso Str. Nuova, 65, 27100 Pavia, Italy; 2 Unit of General Surgery I, Fondazione I.R.C.C.S. Policlinico San Matteo, Viale Camillo Golgi, 19, 27100 Pavia, Italy; 3 Department of Emergency and Trauma Surgery, Scripps Clinic Medical Group, La Jolla, CA USA; 4 General, Emergency and Trauma Surgery Department, Pisa University Hospital, Pisa, Italy; 5 General and Emergency Surgery, Royal Infirmary of Edinburgh, Edinburgh, UK; 6 Ernest E Moore Shock Trauma Center at Denver Health, Denver, CO USA; 7 Division of General Surgery, Rambam Health Care Campus, Haifa, Israel; 8 General Surgery, Monza University Hospital, Monza, Italy; 9 Riverside University Health System Medical Center, Comparative Effectiveness and Clinical Outcomes Research Center - CECORC, Claremont, CA USA; 10 Cambridge Colorectal Unit, Addenbrooke’s Hospital, Cambridge, UK; 11 Departments of Surgery and Critical Care Medicine, University of Calgary, Calgary, Canada; 12 Department of Surgical Sciences and Advanced Technologies, General Surgery Unit, Cannizzaro Hospital, Catania, Italy; 13 Department of Surgical Sciences, Nelson R Mandela School of Clinical Medicine, University of KwaZulu-Natal, Durban, 4001 South Africa; 14 Trauma and Burns Services, Inkosi Albert Luthuli Central Hospital, Mayville, 4058 South Africa; 15 Division of General Surgery, School of Medicine, Istanbul Medeniyet University, Istanbul, Turkey; 16 Division of Surgery and Interventional Science, Hampstead Campus, University College London, London, UK; 17 Department of Surgery, Harborview Medical Centre, University of Washington, Seattle, USA; 18 II Cátedra de Clínica Quirúrgica, Hospital de Clínicas, Universidad Nacional de Asunción, San Lorenzo, Paraguay; 19 Department of Surgery, Erasmus University Medical Centre, Rotterdam, The Netherlands; 20 Department of Surgery, Amsterdam University Medical Center, University of Amsterdam, 1105AZ Amsterdam, The Netherlands; 21 The Research Office, College of Medicine and Health Sciences, United Arab Emirates University, Al-Ain, UAE; 22 Los Angeles County + USC Medical Center, 2051 Marengo Street, Room C5L100, Los Angeles, CA 90033 USA; 23 Department of General Surgery, Royal Perth Hospital, University of Western Australia, Perth, Australia; 24 Department of Surgery, University Hospital Centre Zagreb, Zagreb, Croatia; 25 Division of General Surgery, Department of Biomedical Science for Health, I.R.C.C.S. Ospedale Galeazzi – Sant’Ambrogio, Milan, Italy; 26 Division of Trauma, Emergency Surgery, and Surgical Critical Care, Massachusetts General Hospital and Harvard Medical School, Boston, MA USA; 27 Department of Surgery, Macerata Hospital, Macerata, Italy; 28 Unit of General Surgery, San Benedetto del Tronto Hospital, av5 Asur Marche, San Benedetto del Tronto, Italy; 29 Department of Surgery, Radboud University Medical Center, Nijmegen, The Netherlands; 30 General Surgery Unit, ASST Vimercate, Via Santi Cosma E Damiano, 10, 20871 Vimercate, Italy; 31 Department of Management, Università Ca’ Foscari, Dorsoduro 3246, 30123 Venezia, Italy; 32 Department of General and Thoracic Surgery, University Hospital of Giessen, Giessen, Germany; 33 Department of Digestive and Emergency Surgery, S. Maria Hospital Trust, Terni, Italy; 34 Trauma Department, University of California, Davis, Sacramento, CA USA; 35 Government Gousia Hospital, Srinagar, India; 36 Department of Emergency Surgery, City Hospital, Mozyr, Belarus; 37 Donegal Clinical Research Academy Emergency Surgery Outcome Project, Letterkenny University Hospital, Donegal, Ireland; 38 Cowley Shock Trauma Center at the University of Maryland, Baltimore, MD USA; 39 Department of Surgery, MetroHealth Medical Center Campus, Case Western Reserve University, Cleveland, OH 44109 USA; 40 Third Department of Surgery, Attikon University Hospital, 15772 Athens, Greece; 41 Department of Surgical Science, Cagliari State University, Cagliari, Italy; 42 Department of Emergency Surgery, Centre Hospitalier Intercommunal de Villeneuve-Saint-Georges, Villeneuve-Saint-Georges, France; 43 Virginia Commonwealth University, Richmond, VA USA; 44 Department of Surgery, Tianjin Nankai Hospital, Nankai Clinical School of Medicine, Tianjin Medical University, Tianjin, China; 45 Department of Surgery, Sackler School of Medicine, Tel Aviv University, Tel Aviv, Israel; 46 Division of Trauma and General Surgery, Department of Surgery, University of Pittsburgh Medical Center, Pittsburgh, PA USA; 47 Denver Health Medical Center, University of Colorado Anschutz Medical Center, Aurora, CO USA; 48 Medical School, National and Kapodistrian University of Athens (NKUA), Athens, Greece; 49 Fondazione Policlinico Universitario A.Gemelli IRCCS, Università Cattolica, Rome, Italy; 50 Trauma Center and Emergency Surgery, ASST Grande Ospedale Metropolitano Niguarda, Milan, Italy; 51 Department of General Surgery, Albury Hospital, Albury, Australia; 52 Department of Trauma, Hand and Reconstructive Surgery, University Hospital Frankfurt, Frankfurt, Germany; 53 Department of Anesthesia and Intensive Care, Parma University Hospital, Parma, Italy; 54 Anesthesia and Intensive Care Unit, Ospedale M Bufalini, Cesena, Italy; 55 Service de Chirurgie Digestive et Hépato-Bilio-Pancréatique, Hôpital Henri Mondor, Université Paris Est, Créteil, France; 56 Division of General Surgery, I.R.C.C.S. Ospedale Galeazzi-Sant’Ambrogio, University of Insubria, Varese, Italy; 57 Medical College of Wisconsin, Milwaukee, WI USA; 58 AI Medica Hospital Center / Immanuel Kant Baltic Federal University, Kaliningrad, Russia; 59 Faculty of Health Sciences, Valencian International University (VIU), Valencia, Spain; 60 Department of General Surgery, Military Teaching Hospital, Hôpital Principal Dakar, Dakar, Senegal; 61 Hamad General Hospital, Doha, Qatar; 62 Department of Surgery, Tbilisi State Medical University, Tbilisi, Georgia; 63 RIMU/University Hospital St George, Plovdiv, Bulgaria; 64 Department of Orthopedic Surgery and Neurosurgery, Denver Health Medical Center, University of Colorado School of Medicine, Denver, CO USA; 65 Trauma Service, Auckland City Hospital, Auckland, New Zealand; 66 Tan Tock Seng Hospital, Singapore, Singapore; 67 Department of General y Digestive Surgery, “Dr. Balmis” Alicante General University Hospital, Alicante, Spain; 68 Faculty of Health Sciences, University of Buea, Buea, Cameroon; 69 College of Medicine, University of Arizona, Tucson, AZ USA; 70 Service de Chirurgie Digestive, Centre Hospitalier Universitaire Grenoble Alpes, Grenoble, France; 71 Department of Traumatology, John Hunter Hospital, University of Newcastle, Newcastle, NSW Australia; 72 Bunbury Hospital, Bunbury, WA 6230 Australia; 73 Acute Surgical Unit, Department of General Surgery, Fiona Stanley Hospital, Murdoch, WA Australia; 74 State Major Trauma Unit, Royal Perth Hospital, Wellington Street, Perth, Australia; 75 General Surgery, Monash Medical Centre, Monash Health, Melbourne, VIC Australia; 76 Department of Colorectal Surgery, Logan Hospital, Meadowbrook, QLD Australia; 77 Maitland Private Hospital, East Maitland, Newcastle, NSW Australia; 78 Chris Hani Baragwanath Hospital, Soweto, South Africa; 79 Nambour Selangor Private Hospital, Sunshine Coast University Private Hospital, Birtinya, QLD Australia; 80 Sir Charles Gairdner Hospital, Nedlands, WA Australia; 81 Department of Surgery, Nepean Hospital, Penrith, NSW 2751 Australia; 82 Departamento de Cirugía, Universidad Autónoma de Barcelona, Barcelona, Spain; 83 Department of General Surgery, Nepean Hospital, Sydney, NSW Australia; 84 Department of Trauma, Westmead Hospital, The University of Sydney, Sydney, NSW Australia; 85 Division of Trauma, Siriraj Hospital, Bangkok, Thailand; 86 General and Emergency Surgery, ASST Papa Giovanni XXIII, Bergamo, Italy; 87 General Surgery, Augusta Hospital, Augusta, Italy; 88 Acute Care Surgery Unit, Department of Surgery and Trauma, Maurizio Bufalini Hospital, Cesena, Italy

**Correction: World Journal of Emergency Surgery (2023) 18:42** 10.1186/s13017-023-00511-w

In the original version of this article [1], the given and family names of Adriana Toro were incorrectly structured. The name was displayed correctly in all versions of the publication.

The original article has been corrected.
